# The Contribution of NMR Spectroscopy in Understanding Perovskite Stabilization Phenomena

**DOI:** 10.3390/nano11082024

**Published:** 2021-08-08

**Authors:** Federica Aiello, Sofia Masi

**Affiliations:** 1 National Research Council, Institute for Chemical and Physical Processes (CNR-IPCF), Via G. Moruzzi, 1, 56124 Pisa, Italy; federica.aiello@cnr.it; 2 Institute of Advanced Materials (INAM), Universitat Jaume I (UJI), Avenida de Vicent Sos Baynat, s/n, 12071 Castellón de la Plana, Spain

**Keywords:** perovskite, nuclear magnetic resonance, stability, characterization, bulk, cation, nanocrystals, halide, interactions, ligands, structure, solutions, films, dynamics

## Abstract

Although it has been exploited since the late 1900s to study hybrid perovskite materials, nuclear magnetic resonance (NMR) spectroscopy has only recently received extraordinary research attention in this field. This very powerful technique allows the study of the physico-chemical and structural properties of molecules by observing the quantum mechanical magnetic properties of an atomic nucleus, in solution as well as in solid state. Its versatility makes it a promising technique either for the atomic and molecular characterization of perovskite precursors in colloidal solution or for the study of the geometry and phase transitions of the obtained perovskite crystals, commonly used as a reference material compared with thin films prepared for applications in optoelectronic devices. This review will explore beyond the current focus on the stability of perovskites (3D in bulk and nanocrystals) investigated via NMR spectroscopy, in order to highlight the chemical flexibility of perovskites and the role of interactions for thermodynamic and moisture stabilization. The exceptional potential of the vast NMR tool set in perovskite structural characterization will be discussed, aimed at choosing the most stable material for optoelectronic applications. The concept of a double-sided characterization in solution and in solid state, in which the organic and inorganic structural components provide unique interactions with each other and with the external components (solvents, additives, etc.), for material solutions processed in thin films, denotes a significant contemporary target.

## 1. Introduction 

**Scheme 1:**
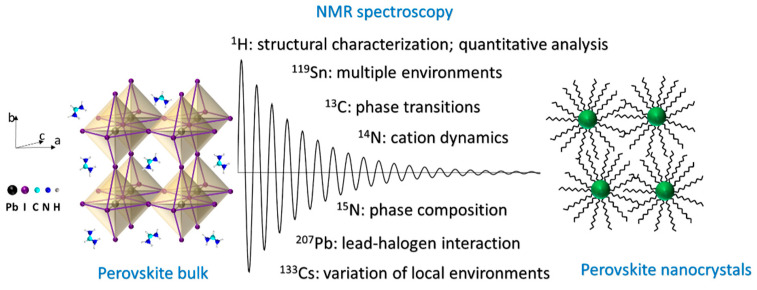
From left to right: sketch of the formamidinium perovskite structure in its cubic crystal phase; NMR tools for perovskite characterization; perovskite nanocrystals with the inorganic core and organic coordinating ligands.

Sustainable photovoltaic applications are evolving from silicon to perovskite materials, where a perovskite with 25.5% efficiency outperforms thin-film silicon solar cells (21.2%), and its efficiency is further increased to 29.5% in tandem devices of perovskite/c-Si [1]. In the common perovskite photovoltaic, the bulk iodide perovskite is pointed out as the best compromise between the band gap (1.57–1.48 eV) to absorb all light and easy synthesis [2]. On the other hand, perovskites have several useful applications, among them light-emitting diodes (LEDs), for which external quantum efficiencies of 20% for green and red emitters and 9% for blue emitters were obtained [3], and photocatalysis [4]. The advantage of the perovskite structure (Scheme 1) is its versatility: it is obtained with different atoms and with different dimensionalities (nanocrystal 0D structure; layered perovskites of the general formula (RNH_3_)_2_A_n−1_B_n_X_3n+1_: n = 1, pure 2D layered; ABX_3_, 3D structure) [5], and it can be mixed with other components in solution [6]. The interplay between compositional engineering, in which the organic–inorganic cation A (methylammonium MA^+^, formamidinium FA^+^, guanidinium Gua^+^ [7], Cs^+^, phenethyalmmonium PEA^+^ [8], polycyclic aromatic [9], secondary ammonium cations [10], etc.), the metal B (Pb^2+^, Sn^2+^, Ge^2+^, etc.) and the halide X (I^−^, Br^−^, Cl^−^) are mixed together, and dimensionality engineering allows for tuning the optical (the band gap is tuned along the complete visible spectra from 1.48 to 3.1 eV) [11] and electrical properties, along with the structural stability and enhanced device efficiencies [12]. In particular, the thermodynamic stability of the polymorphic perovskite for ABX_3_ compounds is predicted with the Goldschmidt tolerance factor, *t*, and the octahedral factor, *μ*:

(1) $$t\;=\;\frac{r_{A}+r_{X}}{\sqrt{2\left(r_{B}+r_{X}\right)}}\;\mathrm{and}\;\mu \;=\;\frac{r_{B}}{r_{X}}$$

where *r_A_* and *r_B_* are the ionic radius of the A and B site cations, respectively, and $r_{X}$ is the ionic radius of the anion. *t* and *μ* are derived from geometric constraints that indicate when the perovskite structure is a possible structure that can be formed. However, these constraints do not necessarily indicate when the perovskite structure adopts the ground-state structure and forms in the stability region, found for 0.875 < *t* < 1.059 and 0.414 < *μ* < 0.732 [13]. To improve the stability, 2D and 0D perovskites have been developed, in which the use of a long hydrophobic aliphatic chain cation A and the increased surface energy lead to less vulnerable structures [14]. 

Enhancement of thermodynamic and moisture stability is perceived as a determining step to boost the perovskite technology’s commercialization; therefore, we need to characterize the material at the atomic level to shine light on the stabilization mechanisms [15].

To investigate the perovskite properties at the atomic level, an array of structural, morphological and spectroscopic techniques is ordinarily used [16]; among them, in this review, we will focus on nuclear magnetic resonance (NMR) spectroscopy as a remarkable technique to entirely characterize the material and the whole system in which it is involved, such as solvents, additives and synthetic components [17,18]. Both solution and solid-state (ss) NMR techniques provide useful information for the characterization of the material and for the elucidation of intermolecular interactions that affect the perovskite stability and the device’s final properties. Solution NMR mainly focuses on the investigation of the interactions between the bulk perovskite precursors and the additives used for improving the film morphology, as well as on the interactions at the surface of perovskite nanocrystals (PNCs) with the organic ligands (Scheme 1); furthermore, this technique allows monitoring ligand exchange processes and evaluating the system stability over time and/or in stressed conditions. On the other hand, ssNMR is exploited for characterizing dried samples and powders, exploring the perovskite phases and correlating the observed properties with the geometry of the crystal structure, as well as for extracting information about the cation dynamics.

NMR spectroscopy offers multiple tools (Scheme 1) for performing a thorough analysis of perovskites; local parameters, such as the chemical shift (δ, usually expressed in ppm), are sensitive to any variation affecting the chemical environment of the nucleus under investigation. Global NMR parameters, such as the diffusion coefficient (D), provide information at a molecular level and can help in estimating the extent of the interaction and how it affects the whole molecule. 

The most useful solution NMR experiments for perovskite characterization are as follows: (i) One-dimensional ^1^H spectroscopy, where changes in the lineshape, linewidth and/or position of proton resonances are indicative of an interaction between, for instance, the organic perovskite precursor and the additive; the experiment, moreover, allows a quantitative evaluation of the sample composition, the identification of impurities and the monitoring of the stability over time and/or under stressed conditions [19,20]. (ii) Diffusion-ordered spectroscopy (DOSY), where variations in the diffusion coefficient are a consequence of interactions involving species with different molecular sizes [21,22]. (iii) ^1^H spin-lattice (T_1_) and spin-spin (T_2_) relaxation measurements, which are sensitive to the variation in the molecular motion consistent with the occurrence of an interaction [17]. (iv) ^1^H-^1^H and ^1^H-^13^C 2D maps for structural characterization and for detection of dipolar intermolecular interactions [23].

Regarding ssNMR, it provides support in the characterization of perovskites via the following: (i) ^1^H analysis, exploited for the determination of the organic cation relative ratio and the analysis of decomposition processes [24,25]. Furthermore, the proton T_1_ is sensitive to phase transitions [26,27]. (ii) ^13^C analysis, for detecting the incorporation of additives within the perovskite framework [28], for elucidating the phase composition at low temperatures [29] and for phase transition identification [30]. Both ^1^H and ^13^C spectra recorded at variable temperatures provide information useful for symmetry evaluation [31]. (iii) ^2^H and ^14^N analysis, to probe the dynamics [32,33,34]. (iv) Exploiting ^207^Pb sensitivity as a probe for detecting alterations in the local electronic structure, coordination environment, ligand electronegativity and temperature [35,36,37]. (v) Analyzing the signal linewidth (dependent on T_2_) to evaluate the nature of the translational motion experienced by the nucleus under investigation.

In this review, we outline the use of NMR spectroscopy to discriminate stable perovskite bulk and nanocrystals (see Scheme 1). We look at particular NMR experiments in solution and in solid state carried out to obtain findings regarding this important aspect, by showing the methodologies developed to relate the structural improvement to the enhanced stability.

## 2. Bulk Perovskites

### 2.1. Solution NMR

Additive engineering was one of the first strategies exploited to improve the quality and the stability of perovskite films [38]. Several organic molecules have been selected to passivate intrinsic defects and/or to confer a certain hydrophobicity to the hygroscopic perovskite material [39]. Organic molecules or polymers are able to passivate defects by ion immobilization, by coordinating Pb ions or by forming hydrogen bonds with the organic cation of perovskites [40]. The “soft” nature of perovskites is, in fact, characterized by ion conduction and ion migration [41], with the drawback of the instability of the perovskite, especially under operation conditions (light, moisture, heat) [42], as well as hysteresis in the devices due to the ion vacancies acting as charge traps [43]. Therefore, the use of additives firstly improves the crystal growth [44], and then the device performances and the long-term stability [45]. However, not all additives are able to blend with the perovskite precursors with the same good effects. Usually, this is due to the different solubility [46] in the solvents commonly employed in perovskite preparation (i.e., dimethylformamide, dimethylsulfoxide, *N*-methylpyrrolidone) [47], and the extent of the interaction, which could hinder or not affect, at all, perovskite formation if too strong or too weak, respectively. In this context, NMR spectroscopy aids in foreseeing the best combination of perovskites and additives by investigating the organic components of the blend, as described below.

#### 2.1.1. Perovskite-Polymer Interactions

**Figure 1:**
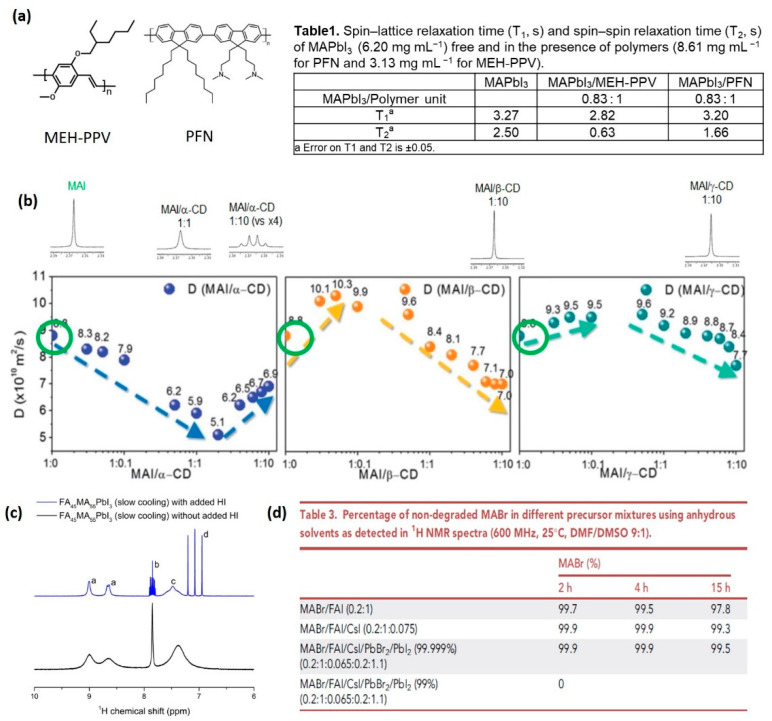
(**a**) Left: chemical structures of MEH-PPV and PFN; right: spin-lattice (T_1_, s) and spin-spin (T_2_, s) relaxation times of MAPbI_3_ free and in the presence of PFN and MEH-PPV. Adapted with permission from Sofia Masi et al. 2015. Copyright 2015 Royal Society of Chemistry. [17]. (**b**) Top: expansion of the low-frequency spectral region of ^1^H NMR spectra of MAI in mixtures with α-CD, β-CD and γ-CD; bottom: plots showing the variation in the diffusion coefficient (D) of MAI during titration with CDs. Reprinted with permission from ref. [21]. Copyright 2021 Royal Society of Chemistry. (**c**) ^1^H NMR spectra of FA_45_MA_55_PbI_3_ spiked with HI (top) and without added HI (bottom). (*a*) Protons of the FA amine groups, (*b*) CH of FA, (*c*) NH_3_^+^ of MA, (*d*) NH_4_^+^. Reprinted with permission from ref. [60]. Copiright 2021 American Chemical Society (**d**) Percentage of non-degraded MABr in different precursor mixtures detected in ^1^H NMR spectra. Reprinted with permission from ref. [61]. Copyright 2021 Cell Reports Physical Science.

Polymeric materials proved to be excellent templating agents for controlling and improving the growth of perovskite films [48]. In 2015, we reported a study aimed at understanding the nature of the interactions involving polymeric additives and perovskite precursors (CH_3_NH_3_I and PbI_2_) in solution [17]. An acid-base equilibrium between the polymer and the methylammonium cation (Figure 1a) was found to be the driving force of the formation of smooth films. The NMR investigation, in this case, was based on ^1^H relaxation measurements rather than diffusion ones; as a matter of fact, the latter could be misinterpreted due to the self-aggregating tendency of perovskite precursors [49]. The interaction was firstly investigated by keeping the precursor/polymer weight ratio constant; under these conditions, only MEH-PPV (poly[2-methoxy-5-(2-ethylhexyloxy)-1,4-phenylenevinylene]) caused a significant variation in the relaxation parameters. The decrease observed for both T_1_ and T_2_ of MAPbI_3_ protons indicates the occurrence of an interaction between the cation and the polymer, which affects the perovskite motion by slowing it down. When the ratio of precursor/number of polymer units, rather than the weight ratio, is kept constant, the interaction with PFN (poly[(9,9-bis(3′-(*N*,*N*-dimethylamino)propyl)-2,7-fluorene)-*alt*-2,7-(9,9-dioctylfluorene)) is also revealed; compared to MEH-PPV, the interaction is weaker, mainly affecting the spin-spin relaxation parameter (Figure 1a). These results suggest that NMR relaxation measurements in solution can be used as a probe for checking the perovskite-additive interaction, correlating a decrease in the relaxation time(s) with a better tendency for the basic functional group to form hydrogen bonds, resulting in a controlled growth. The consequent homogeneous morphology culminates in a more stable thin film. Yet, attention must be paid to the ratio between the perovskite precursors and the polymer units, rather than the weight ratio, which is the most direct approach, used as a first attempt in the study described in [17].

Further studies confirmed that the nature of the interaction between the perovskite precursors and the polymeric additive is paramount to improve the film properties. By simply recording the proton spectra of the pure compounds and the corresponding mixtures with the perovskite, it is easy to distinguish between a weak perovskite-polymer interaction and a strong one. The former can only determine changes in the chemical shift and/or the linewidth of MA^+^ NMR signal(s) [50], while the latter leads to the formation of new species with a consequent disappearance of the cation characteristic proton resonances when precipitation occurs [51]. It is important to underline that the strength of the interaction can be influenced by the synthetic approach taken during sample preparation that could favor the occurrence of strong interactions (ionic-type bonding) over weak ones. 

#### 2.1.2. Solubility Enhancers for Perovskite Precursors

The presence of polymers, despite improving film growth, scarcely contributes to enhancing perovskite crystallinity; this factor is strongly affected by the different solubility of MAI (methylammonium iodide) and PbI_2_, the former being higher than the latter, and the amorphous nature of the polymer cannot provide epitaxial growth propagation nor a supramolecular order. We recently tested native cyclodextrins (CDs) as solubility promoters for the two precursors [21]; the increase in the solubility of the inorganic moiety (PbI_2_) was detected via UV-Vis absorption, whereas NMR was chosen for studying the interactions involving MAI and the macrocycles with different size cavities (α, β and γ in ascending order). Cyclodextrins were chosen because of their well-known ability to establish host-guest interactions with organic molecules: the guest, according to its functional groups, interacts with the hydrophobic inner cavity of the CD and/or with its hydrophilic external surface, decorated by –OH groups [52,53,54,55].

In this study, a strong interaction (complexation) between MAI and α-CD was revealed: the singlet belonging to the MAI methyl group broadens upon addition of the host, changing to a quartet when an excess is added (Figure 1b). Weaker interactions occur with β-CD and γ-CD, with no significant variations in the shape or sharpness observed during the titration experiments. 

The different nature of the interactions with the three cyclodextrins was confirmed with the analysis of the MAI diffusion coefficient (D) in the mixtures (Figure 1b). In samples containing β-CD or γ-CD, D increases at the first points of the titration (MAI in excess), highlighting the interfering effect of the macrocycles on the self-assembly propensity of the organic salt; with a large excess of the hosts, complexation is the driving force, resulting in a decreasing tendency of D. The strong interaction with α-CD causes an opposite trend, suggesting the predominance of inclusion phenomena in the first part of the titration due to a smaller inner cavity of the host, which could better accommodate the guest (Figure 1b). The strong interaction between the organic cation and α-CD hinders the perovskite formation, while the weaker one with β-CD is suitable for improving the crystallinity, the stability and the optoelectronic properties of the material.

#### 2.1.3. Stability of Mixed Cation Perovskite Solutions

Solution NMR spectroscopy is also successfully employed in the characterization of perovskite systems containing mixed cations. As a matter of fact, compositional engineering is the most important and diffused strategy for perovskite preparation, as it leads to recording solar cells’ efficiency and stability. Moreover, by mixing cations, the stability of the metastable perovskites, such as FAPbI_3_ and CsPbI_3_, improves, and the thermal stability of MAPbI_3_ is enhanced. The contribution of solution NMR spectroscopy in this area relies on the opportunity to easily quantify the organic cations’ molar ratio via ^1^H NMR, by integration of the proton signals corresponding to the different species [56,57,58]. Such analysis also proved to be reliable in detecting and quantifying the cation transport between layers in mixed cation perovskites (i.e., methylammonium/ethylammonium) on the basis of the bifacial stamping process [59]. In the analysis of FA_x_MA_1−x_PbI_3_ crystals, ^1^H NMR investigation allows monitoring the degradation of formamidine to ammonia, related to the cooling rate used in the synthetic procedure (Figure 1c). Recently, a protocol was proposed for reliable quantification of FA_x_MA_1−x_PbI_3_ samples, in which hydroiodic acid is added to improve the resonances’ linewidth and guarantee accurate results [60]. The real cation ratio in the perovskite, often different from the nominal one, is, in fact, vital to understand the interrelationship between material properties.

Triple-cation mixed halide perovskite precursors have recently been deeply investigated via ^1^H NMR, in order to find a rationale behind the worsening of device performances over time [61]. Quantitative analysis performed on a mixture of cation precursors indicated that the MA^+^ and FA^+^ degradation products emerge in a lower percentage when Cs^+^ is present (Figure 1d), thus highlighting its stabilizing effect on the organic cations; on the contrary, the presence of water and/or basic impurities is responsible for a faster degradation. The NMR investigation, moreover, allowed a full characterization of the detected degradation products, by exploiting a combination of homo- (^1^H-^1^H) and heteronuclear (^1^H-^13^C) 1D and 2D experiments. Such analysis made the identification, as by-products, of trans/cis stereoisomeric species, resulting from a reaction of the nucleophilic addition involving methylamine and FA^+^, possible [62].

### 2.2. Solid-State NMR

The enormous potentialities of solid-state (ss) NMR spectroscopy in the characterization of perovskite materials have been exploited since the late 1980s [33,63,64,65]; in the last ten years, the application of this spectroscopic technique contributed to a better understanding of the material properties. The remarkable versatility of this analytical technique is related to the fact that all perovskite elements can be observed: the cation (via, for instance, ^1^H, ^2^H, ^13^C, ^14^N, ^133^Cs NMR), the metal and the halide, and precise information about the structure and dynamics can be obtained. Franssen and Kentgens published, in 2019, an exhaustive overview of the applications of ssNMR spectroscopy in bulk perovskite characterization [66], which was followed by an article by Kovalenko in 2020 that complemented the overview by presenting the potentialities of nuclear quadrupolar resonance (NQR) in ^79/81^Br and ^127^I analysis [67]. Here, we update these reviews with the most recent publications focusing on improving film stability and on the analysis of layered systems. Layered perovskites improve the stability of solar cells if compared to 3D perovskites; however, the mechanism remains unclear. The most important types of 2D perovskites are the Dion–Jacobson (DJ) and Ruddlesden–Popper (RP) crystal structures, obtained by different techniques [68]. An additional larger organic cation is introduced as a spacer, isolating the inorganic metal halide octahedral; by modifying the spacer, the photo-physical properties are tuned. The RP perovskites have a typical van der Waals layered crystal structure. Interestingly, this particular type of perovskite has naturally integrated quantum well structures exhibiting quantum confinement effects [69]. The improved environmental stabilities, large exciton binding energies and high quantum yields make this family of perovskites a promising candidate for solar cells and LEDs [70]. 

#### 2.2.1. Organic Molecules for Improving Formamidinium Perovskite Stability

The use of compositional engineering [71] or external additives [72] is vital to obtain a photoactive stable crystal α-phase, due to its polymorphic nature [14]. With investigations at the atomic level, we gain information about the role of the stabilizer, particularly concerning how and why it improves the perovskite stability. Advanced crystallographic characterization discerns that a more symmetric orientation of the perovskite cation leads to improved perovskite crystallinity and, in turn, stability; therefore, in this view, ssNMR is an elegant approach to corroborate it [21]. For instance, ssNMR spectroscopy was successfully employed in the investigation of the interaction of a formamidinium-based perovskite with 5-ammonium valeric acid iodide (AVAI) [73] and 1-adamantylammonium halides [74]. 

**Figure 2:**
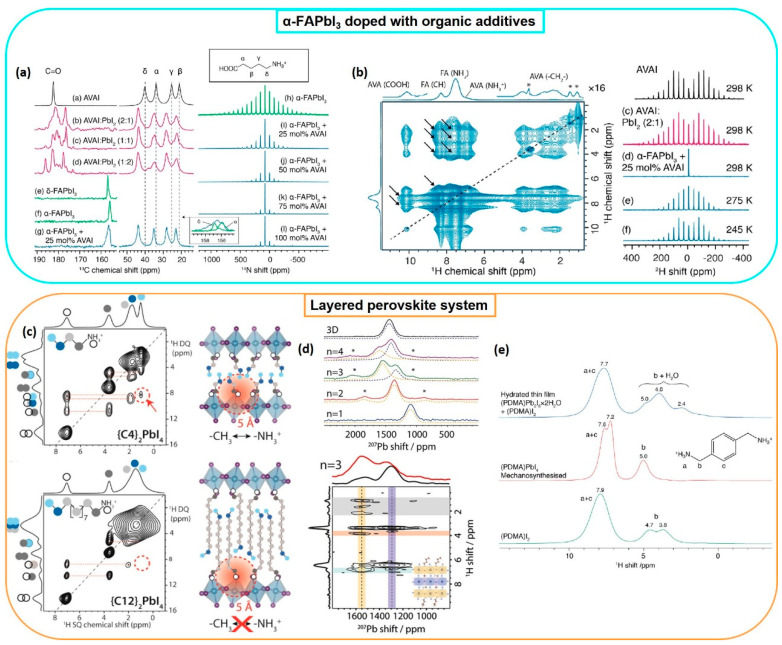
(**a**) Left: ^1^H−^13^C CP MAS spectra of AVAI and AVAI:PbI_2_ at different molar ratios; right: ^14^N MAS NMR spectra of α-FAPbI_3_, and α-FAPbI_3_ doped with different amounts of AVAI. (**b**) Left: ^1^H-−^1^H spin diffusion measurement, where the arrows indicate the intermolecular interactions between CH and NH_2_ of FA and AVA protons; right: ^2^H MAS spectra of neat AVAI, (c) AVAI:PbI_2_ and α-FAPbI_3_ + 25 mol% AVAI at (d) 298 K, (e) 275 K and (f) 245 K. Reprinted with permission from ref. [73]. Copyright 2019 American Chemical Society. (**c**) Two-dimensional ^1^H DQ−SQ NMR correlation spectra of (C4)_2_PbI_4_ and (C12)_2_PbI_4_. Reprinted with permission from ref. [78]. Copyright 2021 American Chemical Society. (**d**) Top: ^207^Pb Hahn-echo spectra of 2D BA_2_MA_n−1_Pb_n_I_3n+1_ and 3D MAPbI_3_ perovskite; bottom: ^1^H-detected ^207^Pb → ^1^H spectra for BA_2_MA_n−1_Pb_n_I_3n+1_ (n = 3). Spinning sidebands are marked by asterisks (*). Reprinted with permission from ref. [79] Copyright 2021 American Chemical Society. (**e**) ^1^H NMR spectra of (PDMA)I_2_, mechanosynthesized (PDMA)PbI_4_ and scraped thin films of (PDMA)PbI_4_ following hydration. Reprinted with permission from ref. [80]. Copyright 2021 American Chemical Society.

The analysis of ^13^C cross-polarization (CP) magic angle spinning (MAS) NMR spectra of the organic molecule in the presence of PbI_2_ reveals a remarkable variation in the spectral profile, whereas a shift at higher frequencies is observed in the presence of the perovskite (Figure 2a). When mixed with the stabilizer, the perovskite resonance is broader, indicating a different environment caused by the interaction. ^1^H-^1^H spin diffusion (SD) measurements allow the detection of intermolecular dipolar interactions, further confirming the atomic-scale interaction between the compounds and the perovskite lattice (Figure 2b). The cation dynamics is instead elucidated by exploiting ^2^H and ^14^N, both quadrupolar nuclei. The width narrowing of spinning sidebands in ^14^N MAS spectra, caused by the quadrupolar interaction, indicates a higher reorientation symmetry of the perovskite cation, and hence a more symmetric environment upon addition of the stabilizer. Analysis of dynamics via ^2^H NMR indicated that it has a high freedom in motion that is dynamically disordered when embedded into the perovskite; nevertheless, the resultant doped perovskite is more stable, pointing out that the control and the low amount of the disordered cation in the perovskite lattice do not influence the stability of the formamidinium perovskite.

The same research group recently focused on the effect of benzylammonium iodide on the stability of formamidinium-based perovskites [75]. Interestingly, the analysis of ^14^N spinning sidebands shows an increase in the full width at half maximum upon addition of the stabilizer, which is hence responsible for an alteration in the cubic symmetry, without reducing the dimensionality of the perovskite.

Analogously, molecular modulators endowed with ammonium (–NH_3_) and thiol (–SH) groups used as crystallinity and stability enhancers were characterized [76]. In this case, the effectiveness of the –NH_3_^+^ group in reducing lattice defects is combined with the size increase in both perovskite grains and crystals and with the formation of an intermediate SN–PbI_2_ adduct triggered by the –SH group, thus making these multifunctional derivatives very promising for perovskite stability improvement. In perspective, the use of sulfur-based organic additives will be considered for metastable perovskite stabilization, also due to the great interest created by the use of external stabilizer additives such as PbS quantum dots [72] or nanoplatelets [77], which are able to epitaxially propagate the crystal lattice parameter while creating chemical bonds between the Pb and S atoms.

Anion passivation strategies have proven to be efficient in improving perovskite stability and crystallinity; recently, the effects of formate were investigated by Jeong et al. [78]. ^13^C CP MAS analysis performed at low temperature shows that the anion carboxylic resonance significantly broadens when the compound is added to the perovskite, suggesting an interaction between HCOO^−^ and the undercoordinated metal. The resonance in the ^207^Pb MAS NMR spectra, on the contrary, is not affected by the addition of formate, indicating that the interaction passivates the vacancies without involving iodide replacement in the perovskite lattice, which would have affected the metal environment and, consequently, its NMR spectrum. Through this method, solar cells reached a certified 25.2% of efficiency and an improved thermal and operational stability due to a controlled halide passivation, especially at the grain boundaries, as demonstrated by ssNMR spectroscopy.

#### 2.2.2. Layered Systems

Alkylammonium and aromatic spacer cations were recently analyzed by Reddy et al., who focused on their arrangement between the layers in RP 2D perovskites [79]. The question concerns whether the dynamic interactions between the spacer molecules and the inorganic octahedra have an impact on the photoluminescence quantum yield (PLQY) and carrier lifetimes; both these parameters are correlated with the presence of defects and, hence, with the stability of the material. The mechanism triggered by thermal reorientation is still under investigation, and ssNMR was exploited to partially address this issue by developing this analytical protocol: (1) ^1^H-^1^H double-quantum–single-quantum (DQ-SQ) experiments were performed on spacers with different lengths, indicating a trans-orientation of the spacers (Figure 2c), with the organic chains extended; (2) local mobility was investigated via ^13^C CP MAS experiments performed at variable contact times, which revealed a relationship between the group rigidity (related to the signal intensity build-up in the NMR spectrum) and its distance from the metal; (3) comparison of the ^15^N CP MAS build-up kinetics of spacers with different lengths was used to show a link between the chain length and NH_3_^+^ mobility when in proximity to PbI_6_ octahedra. Finally, it was found that bulky aromatic and short alkylammonium spacers are similarly rigid, thus likely leading to similar optoelectronic properties. On the contrary, spacers with linear alkylammonium chains with variable lengths (C4 or C12) result in a more rigid methylene group near the octahedral surface (C4) or within the interlayer (C12). This finding is correlated with the tilting of the octahedra, which would have an effect on the structural stability and on the abovementioned photoluminescence properties. 

Given that the metal environment influences the ^207^Pb NMR chemical shift, it is possible to differentiate between the metal layers located next to the spacer and those in a 3D-like environment [80]; their assignment can be performed by observing ^207^Pb-^1^H correlations, detected via HETCOR (heteronuclear correlation spectroscopy) experiments. In the map reported in Figure 2d, it is possible to distinguish between the outer lead signal, which correlates with the spacer proton resonances, and the inner one, which only correlates with the perovskite small cation. The Pb–halide (average) bond distance is linearly correlated with the isotropic chemical shift observed in the ^207^Pb MAS NMR spectrum; therefore, this parameter could be used for locally describing the metal environment. 

In the evaluation of the impact of moisture on DJ perovskites, ssNMR provides information complementary to X-ray diffraction, UV-Vis spectroscopy and thermogravimetric analysis. In a recent study performed on 1,4-phenylenedimethanammonium (PDMA)PbI_4_, ^1^H MAS NMR spectra of precursors and mechanosynthesized and hydrated perovskites were compared, clearly showing the presence of water signals below 5 ppm in the last sample (Figure 2e) [81]. Despite the strong superimposition between the water signal and the spacer’s methylene protons, water quantification is still possible by subtracting the perovskite contribution from the integral (which can be calculated from the NH_3_^+^ proton signal).

#### 2.2.3. Cesium-Based Perovskites

**Figure 3:**
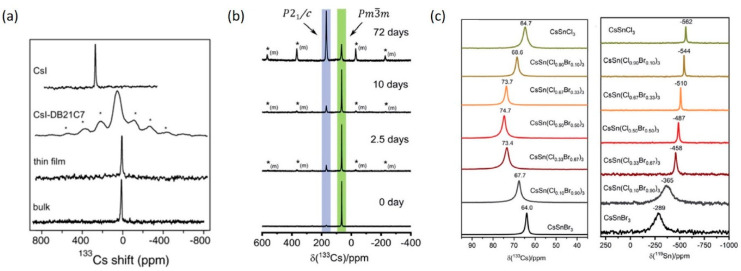
(**a**) ^133^Cs MAS spectra of crystalline CsI, CsI–DB21C7 powder, FAPbI_3_ thin film treated with CsI–DB21C7 (thin film) and a sample of FAPbI_3_ mixed with the CsI–DB21C7 complex (bulk); * indicate ^133^Cs spinning sidebands. Reprinted with permission from ref. [82]. Copyright 2021 Springer Nature. (**b**) Time-dependent ^133^Cs NMR spectra of CsSnCl_3_. The asterisks (*(m)) indicate positions of spinning sidebands for the monoclinic phase. (**c**) Left: room temperature ^133^Cs NMR spectra for CsSn(Cl_1-x_Brx)_3_; right: room temperature ^119^Sn NMR spectra for CsSn(Cl_1-x_Br_x_)_3_. Reprinted with permission from ref. [83]. Copyright 2021 Royal Society of Chemistry.

Crown ethers have been investigated in virtue of their selectivity towards small alkali metal ions (as Cs^+^), which can be exploited to convey the metal within the perovskite film without affecting its stability. It has been recently proven that dibenzo-21-crown-7 (DB21C7) forms host-guest complexes with Cs^+^; such interaction can be detected by analyzing the ^133^Cs NMR spectrum of the host-guest complex Cs–DB21C7 [82]. The coordination to the metal is highlighted by a broad resonance and spinning sidebands, suggesting a less symmetric Cs^+^ environment (Figure 3a). Interestingly, when embodied within the perovskite, the resonance not only gains in sharpness (as a consequence of a more symmetric environment) but its chemical shift is also linearly dependent on the metal concentration, providing a way to quantify the Cs^+^ incorporation.

^133^Cs sensitivity towards variations in the local environment is also exploited in NMR analysis of lead-free perovskites [83]. The transition from the cubic to the monoclinic phase for CsSnCl_3_ is easily monitored over time, as the ^133^Cs NMR resonance of the two forms differs in position and linewidth (Figure 3b). In the same work, it was shown that ^133^Cs can be combined with ^119^Sn in the investigation of a mixed halide CsSn(Cl_1−x_Br_x_)_3_ perovskite. A peculiar dependence on the halogen ratio is found for the ^133^Cs chemical shift (Figure 3c); importantly, the signal linewidth and the absence of significant spinning sidebands suggest a rapid halogen dynamics in the Cs cuboctahedral. On the other hand, the ^119^Sn resonance shifts to lower chemical shifts and decreases in linewidth with increasing Cl content (Figure 3c). These spectral features constitute a powerful set of tools for comparing different synthetic procedures, given that the presence of impurities affects the crystallinity and, therefore, determines detectable differences in the NMR spectra. Analogously, the stability of the perovskite can be monitored over time, and the effect of room temperature oxidation can be estimated on the basis of the by-products formed.

Besides mixing halogens, metals are also combined in order to improve the sample stability and final properties. The local structure of a mixed system such as CsSn_x_Pb_1−x_Br_3_ can be analyzed via ^119^Sn, ^133^Cs and ^207^Pb NMR [84]. The ^133^Cs NMR spectra of CsSnBr_3_ and CsPbBr_3_ are indeed very different, due to the high symmetry experienced by Cs^+^ in the former (single peak in the NMR spectrum) as opposed to the latter (larger linewidth and spinning sidebands). Therefore, the spectrum of a mixed metal perovskite is strongly influenced by the Sn/Pb ratio. ^119^Sn and ^207^Pb resonances have a linear and opposite dependence on the metal ratio: with increasing Pb, the former shifts at higher frequencies and the latter at lower frequencies. The increase in linewidth observed for both nuclei is expected, due to the presence of multiple environments surrounding the metals.

## 3. Nanocrystal Perovskite 

### 3.1. Solution NMR

The interest in the stabilization of PNCs lies in the fact that they have amazing theoretical properties for light harvesting in solar cells (i.e., CsSnI_3_ has the narrowest band gap of 1.3 eV) [85], and, at the same time, they have attracted enormous attention as emitters with a high PLQY, tunable band gap from violet to infrared and narrow emission widths for a high color purity, which can be fabricated from solutions of inexpensive precursors [3]. For instance, the color gamut of displays made by PNCs can cover up to 140% of the National Television System Committee standard and is close to 100% of the new International Telecommunication Union Rec. 2020 standard, making them promising for ultra-high definition displays [86,87].

Although PNCs are more stable than their bulk counterpart [14], the stability of CsBX_3_ is still a big challenge, and it compromises their application in devices. They have a defective structure, negatively affecting the PLQY, related to the essential but, at the same time, labile interaction between the colloidal ligand and the inorganic core [18]. Trap states are usually associated with intrinsic point defects, such as vacancies, interstitials and anti-site occupations. Usually, alternative capping ligands such as didodecyl dimethyl ammonium bromide (DDAB) [32] are introduced by loading them in the synthesis or as post-treatment. However, the ligands are highly labile and easily desorb from the surface during the purification, leading to a high density of dangling bonds at the surface of PNCs [88]. 

Solution NMR spectroscopy, already largely applied in colloidal NC analysis [89], contributes to the characterization of PNCs by: (i) checking the purity and the integrity of the product after the purification steps [90,91], (ii) evaluating the stability over time and the effect of the halide [23], (iii) investigating the capping ligands [18] and their interactions with the ligands themselves, the lattice and/or the precursors [92,93,94,95,96] and iv) providing insights into ligand exchange [97] and self-assembly [98] processes aimed at improving the stability and final device properties.

**Figure 4:**
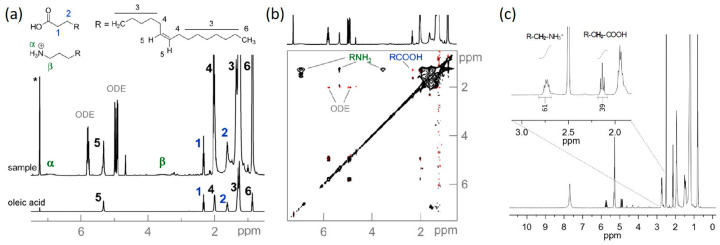
(**a**) ^1^H NMR spectrum of CsPbBr_3_ NCs (sample) and reference spectrum for oleic acid. (**b**) NOESY map of CsPbBr_3_ NCs. Reprinted with permission from ref. [88]. Copyright 2016 American Chemical Society.; (**c**) ^1^H NMR spectrum of Cs_4_PbBr_6_ NCs dissolved in DMSO-d_6_ after the addition of small amounts of protonating agent trifluoroacetic acid for quantification of the ratio between oleylamine and oleic acid. Reprinted with permission from ref. [94]. Copyright 2020 Royal Society of Chemistry.

Capping ligands are usually low-molecular weight (<1000 Da) organic molecules; therefore, their interactions with the NC surface can be successfully monitored via 1D NMR experiments, allowing distinguishing between free- and bound-state ligands [99]. The technique is used to extract information about the binding affinity, providing a tool to compare the efficiency of different ligands; moreover, 2D experiments constitute a useful tool for the observation of ligand-ligand and ligand-solvent interactions. Kovalenko’s group is one of the pioneers in the use of solution NMR spectroscopy for the characterization of PNCs; in 2016, they analyzed the ^1^H NMR spectra of the free capping ligands oleic acid (OA) and oleylamine (OLA) in comparison with the colloidal system of the perovskite CsPbBr_3_ (Figure 4a) [88]. The remarkable increase in linewidth observed for the OLA signals is indicative of the interaction with the perovskite surface; on the contrary, the OA signals are as resolved as in the free ligand spectrum, suggesting that the acid is not bound to the surface, negatively affecting the colloidal stability of the perovskite. The lack of interaction is also confirmed by analyzing the 2D NOESY (nuclear Overhauser effect spectroscopy) map, where negative cross-peaks are detected for OA resonances in contrast to positive cross-peaks for OLA (Figure 4b). This difference is due to the binding of the amine to the perovskite surface, which reduces the speed of its molecular motion (black positive cross-peaks); OA has, instead, more freedom in solution (red negative cross-peaks). Information about the dynamics of the interaction is obtained by analyzing the ligand diffusion coefficients via DOSY experiments and by comparing them to the pure compounds (a decrease in D corresponds to an increase in molecular sizes); moreover, diffusion data are exploited for extrapolating the bound and free molar fractions of the ligand. Interestingly, by combining 1D and 2D data, a deep analysis of the equilibria in which the ligands are involved in solution is carried out, highlighting the role of acid-base interactions on the NC structural integrity and stability after the standard procedure of purification.

Great attention is devoted to synthesis optimization for obtaining PNCs endowed with robust surface passivation. Manna et al. recently explored the conversion of Cs_4_PbBr_6_ to CsPbBr_3_ NCs mediated by poly(maleic anhydride-alt-1-octadecene) (PMAO) [94]. A metal-poor perovskite was characterized via 1D and 2D NMR experiments, and a protocol for the quantification of the capping ligand molar ratio was developed. This protocol involves dissolving the NCs in deuterated DMSO and adding trifluoroacetic acid as a protonating agent able to help the separation in the proton spectra of the ligand resonances (Figure 4c), which can then easily be integrated and quantified. NMR spectroscopy is able to highlight the occurrence of an interaction between PMAO and the ligand oleylamine, thus shedding light on the perovskite phase conversion mechanism and on the pivotal role of the polymer. Moreover, it is observed that CsPbBr_3_ NCs obtained via this alternative route show enhanced stability compared to ligand-capped CsPbBr_3_ synthesized according to the classical procedure (cesium oleate/lead(II) bromide route), attributable to PMAO and its surface interaction with the perovskite.

The acid-base interaction of the ligands (oleic acid and oleylamine) in solution needs to be carefully controlled in order to avoid side products, such as the formation of N-oleyl-oleamide [18]. By ^1^H NMR quantitative analysis, it is found that, more than the presence of by-products, the most important aspect is the ratio between the different species in solution (acid, amine and amide) and the surface dimension of the PNCs, pointing out that the size and, in turn, the optical properties of the perovskite could be easy tuned, overcoming the limitation of the synthetic parameter, such as the temperature limit and the reactant concentrations.

### 3.2. Solid-State NMR

We illustrated how solution NMR spectroscopy is a useful tool in the characterization of PNCs, as different experiments can be run in order to provide information about the colloidal suspension. On the other hand, it is important to evaluate the final sample properties, once dried, and ssNMR is of help in elucidating the ligand interaction with the film, and in characterizing the organic surface.

Monitoring the carboxylic spectral region (around 180 ppm) in the ^13^C CP MAS NMR spectrum of perovskite allows evaluation of the ligand exchange efficiency by assessing whether a carboxylate ligand is bound to the NC surface, as reported by Brown et al. [100]. If the replacing ligand is a phosphonate, such as octylphosphonic acid, ^31^P NMR can be exploited to confirm the interaction with the perovskite, which causes an upfield shift in ligand resonances. The extent of the shielding also provides information on the nature of the ligand, keeping in mind that neutral ligands undergo a smaller variation in their chemical shift with respect to deprotonated ones, which have a stronger interaction with the perovskite metal. Even if not directly coordinated to the PNCs, the hydrogen-bonded inter-ligand network between the groups P–OH and P = O is envisaged as an alternative passivation method to improve the final PNC optical properties.

**Figure 5:**
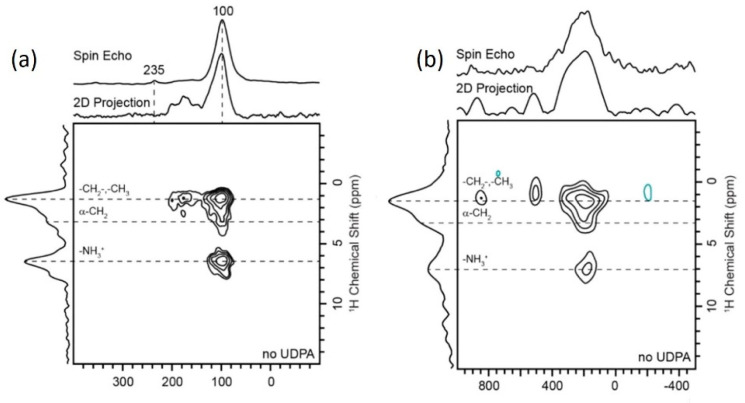
(**a**) ^133^Cs spin echo and 2D 1H → ^133^Cs CP-HETCOR NMR spectra of CsPbBr_3_ QDs without UDPA; (**b**) ^207^Pb spin echo NMR spectra and 2D ^207^Pb → 1H CP-HETCOR of CsPbBr_3_ QDs without UDPA. Reprinted with permission from ref. [101]. Copyright 2020 American Chemical Society.

Rossini et al. very recently characterized the CsPbBr_3_ NC surface via ssNMR; first, they focused on the characterization of ligands (dodecylammonium, oleate and/or 10-undecenylphosphonate UDPA), by strengthening the ^1^H attribution with 2D heterocorrelation experiments [101]. The nature of the NC surface termination, critical to assess the charge trap in the PNCs, was, instead, investigated via ^133^Cs and dipolar ^1^H-^133^Cs CP-HETCOR experiments. The previously mentioned sensitivity of the metal for different environments makes it possible to distinguish, in the ^133^Cs NMR spectrum, the resonance belonging to the species in the NC bulk (centered at 100 ppm) from that on the surface at 170 ppm (Figure 5a). This resonance shows a higher relative intensity in the CP MAS spectrum, confirming its proximity to the organic ligands and the Cs termination.

Similarly, ^207^Pb-^1^H CP-HETCOR experiments highlight the portion of the metal close to the surface and interacting with the proton resonances belonging to the ligands; the absence of significant differences between the spin echo spectrum is an indication that ^207^Pb is mainly in the bulk and not present on the surface (Figure 5b). To rule out any possible interference in Pb detection at the NC surface (due, for instance, to chemical shift anisotropy), ^1^H-^207^Pb correlations are detected by exploiting the DHMQC (dipolar heteronuclear multiple quantum coherence) experiment. Finally, the dipolar coupling between ligands with an ammonium functionality and the metals can be estimated via ^1^H(^207^Pb) S-REDOR (symmetry-based resonance-echo double-resonance) and ^1^H(^133^Cs) RESPDOR (rotational-echo saturation-pulse double-resonance) experiments, which allow estimating the inter-nuclear distances.

## 4. Concluding Remarks and Future Perspectives

Perovskite materials show promising optoelectronic properties and device efficiencies; however, their stability is a drawback for the commercialization of perovskite-based technologies. The instability lies in atomic reasons, as the structure is stabilized if the cation and the inorganic octahedra fit the dimension and the space distribution of the tetragonal/cubic crystal phases. Moreover, defects contribute to the instability and need to be passivated at the atomic level. External factors such as moisture, heat and light worsen and accelerate the transitions to photoinactive crystal phases until the degradation of the precursors. NMR spectroscopy provides tools to characterize perovskite materials and to foresee the best method to stabilize them, but also to follow the changes and the degradation over time. An overview of the nuclei described in the previous sections and of the extracted information is reported in Table 1.

In general, we find that the following evidence, supported by NMR spectroscopy, is correlated with improved stability:

(1) The interaction between perovskite precursors and additives, particularly with polar functional groups. The strength of the interaction is proved and measured by NMR in solution, via investigation of signal shifts in the ^1^H spectra and changes in relaxation measurements (T_1_ and T_2_) and/or in the diffusion coefficients (D). Analysis of ^13^C CPMAS, ^14^N MAS and ^1^H-^1^H spin diffusion measurements in solid samples corroborates the different environments or a more symmetric distribution of the perovskite components in the case of additives. Moreover, ^13^C experiments are a sensitive indicator for phase composition (i.e., yellow/black phase of FAPbI_3_, without/with an additive, respectively).
(2) Use of big organic cations as spacers in the 2D/3D composition. ^207^Pb-^1^H correlations, detected via HETCOR experiments, discriminate between the outer and the inner lead signal in 2D/3D perovskites. In general, the increase in linewidth observed for ^1^H, ^13^C and ^14^N nuclei is expected, if multiple environments are surrounding the metal (i.e., Cs^+^, Pb^2+^, Sn^2+^). However, the introduction of a long alkyl chain cation or an external organic additive could negatively affect the charge transport. Thus, in order to foresee the optimum method and optimum organic additive/cation for the stabilization of the perovskite phase, characterization with isotopic enrichment (^2^H) is needed to clearly define the orientation and the localization of organic cations in the bulk with respect to Pb, and the nature of the interaction with other perovskite elements.
(3) Controlled compositional engineering. ^1^H NMR has an excellent reliability in quantifying the cations in solution, allowing for the identification of their exact molar ratio. Moreover, reactions and side products are detected, and long-term stability is analyzed by exploiting homo- (^1^H-^1^H) and heteronuclear (^1^H-^13^C) 1D and 2D experiments. Despite the great progress with ssNMR for detecting the phase transitions in single-cation perovskites (i.e., the transition from the cubic to the monoclinic phase for CsSnCl_3_), it is crucial to also distinguish the different crystal phases in mixed cation-halide perovskites, in order to explore potential black phases for optoelectronic applications. For this purpose, ^207^Pb experiments provide a tool for investigating the lead-halogen interaction and PbX_6_ symmetry, while the analysis of the cation reorientation with respect to Pb is still difficult to probe quantitatively and needs costly isotopic enrichment (^15^N or ^2^H experiments).
(4) Efficient ligand coordination with the inorganic core of PNCs. ^1^H, NOESY and DOSY experiments provide information about the ligands in free and bound states, providing insights into the ligands ratio and the nature of the chemical bond of the ligand coordinated to the surface, which positively passivate defects and affect the colloidal stability of the perovskite. The introduction of various ligands stabilizes perovskite NCs. However, the mechanism is not completely understood, and more efforts are needed to characterize, at the atomic level, these systems, along with the long-term stability in different solvents.

## Figures and Tables

**Table 1:** List of NMR active nuclei exploited for perovskite characterization.

Nucleus	Spin	NMR Experiment	Information Obtained
^1^H	½	1D spectrum	Quantitative determination of the organic cations ratio Detection of strong and weak interactions with additives Characterization of free and bound organic ligands for nanocrystals Analysis of stability
		T_1_ and T_2_ measurements	Changes in molecular motions due to intermolecular dipolar interactions
		DOSY	Changes in hydrodynamic radius due to complexation/interaction Detection of ligand-nanocrystal surface interaction
		2D experiments (^1^H-^1^H and ^1^H-X)	Detection of intra- and intermolecular dipolar interactions Characterization of impurities Characterization of ligands Detection of ligand-nanocrystal surface interaction
^2^H	1	1D spectrum	Detection of phase transitions via analysis of cation dynamics
^13^C	½	CP MAS spectrum	Detection of interactions with additives Analysis of phase composition Evaluation of ligand exchange in nanocrystals
^14^N	1	1D spectrum	Analysis of spinning sidebands for elucidating cation reorientation dynamics
^207^Pb	½	1D spectrum	Analysis of sample composition in mixed systems Changes at the metal environment (symmetry)
^133^Cs	½	1D spectrum	Changes at the metal environment (symmetry) Detection of phase transitions Detection of impurities Analysis of sample composition in mixed systems
^119^Sn	½	1D spectrum	Detection of impurities Analysis of sample composition in mixed systems

## Data Availability

Not applicable.
